# Behavioural Responses to Thermal Conditions Affect Seasonal Mass Change in a Heat-Sensitive Northern Ungulate

**DOI:** 10.1371/journal.pone.0065972

**Published:** 2013-06-11

**Authors:** Floris M. van Beest, Jos M. Milner

**Affiliations:** 1 Department of Animal and Poultry Science, College of Agriculture and Bioresources, University of Saskatchewan, Saskatoon, Saskatchewan, Canada; 2 Faculty of Applied Ecology and Agricultural Sciences, Hedmark University College, Evenstad, Norway; Université de Sherbrooke, Canada

## Abstract

**Background:**

Empirical tests that link temperature-mediated changes in behaviour (activity and resource selection) to individual fitness or condition are currently lacking for endotherms yet may be critical to understanding the effect of climate change on population dynamics. Moose (*Alces alces*) are thought to suffer from heat stress in all seasons so provide a good biological model to test whether exposure to non-optimal ambient temperatures influence seasonal changes in body mass. Seasonal mass change is an important fitness correlate of large herbivores and affects reproductive success of female moose.

**Methodology/Principal Findings:**

Using GPS-collared adult female moose from two populations in southern Norway we quantified individual differences in seasonal activity budget and resource selection patterns as a function of seasonal temperatures thought to induce heat stress in moose. Individual body mass was recorded in early and late winter, and autumn to calculate seasonal mass changes (*n* = 52 over winter, *n* = 47 over summer). We found large individual differences in temperature-dependent resource selection patterns as well as within and between season variability in thermoregulatory strategies. As expected, individuals using an optimal strategy, selecting young successional forest (foraging habitat) at low ambient temperatures and mature coniferous forest (thermal shelter) during thermally stressful conditions, lost less mass in winter and gained more mass in summer.

**Conclusions/Significance:**

This study provides evidence that behavioural responses to temperature have important consequences for seasonal mass change in moose living in the south of their distribution in Norway, and may be a contributing factor to recently observed declines in moose demographic performance. Although the mechanisms that underlie the observed temperature mediated habitat-fitness relationship remain to be tested, physiological state and individual variation in thermal tolerance are likely contributory factors. Climate-related effects on animal behaviour, and subsequently fitness, are expected to intensify as global warming continues.

## Introduction

Investigating physiological consequences of behavioural choices made by individuals, including altered activity patterns and non-random habitat use [1], [2], is fundamental to ecological theory and improving conservation and management actions [3]. Numerous biotic factors are known to impact individual behaviour, habitat choice and subsequent fitness, such as the spatial distribution and abundance of high quality forage resources [1], human disturbance [4], and predation pressure [5]. The importance of abiotic factors, such as climate, on behaviourally-mediated fitness effects is less well documented despite growing evidence of the influence of temperature on changes in animal behaviour [6], species distributions [7] and population dynamics [8], [9].

The importance of the thermal environment on animal behaviour, and consequently, effects on demography and ecology, has long been recognized in ectotherms [10]. In contrast, most research on warm-blooded species has focussed on direct effects of extreme climatic events on survival and reproduction [9], [11] or indirect effects of temperature on body size through changes in plant phenology and vegetation productivity [12]. Empirical evidence of the impact of contemporary ambient temperatures on changes in behaviour and the effect on individual condition is lacking for free-ranging endotherms.

Although endotherms are able to maintain a relatively constant body temperature as ambient temperature fluctuates, this is energetically costly and expenditure increases dramatically when an individual is outside its thermoneutral zone [13]. Endotherms use a range of thermoregulatory behaviours to limit the effects of ambient temperature on their energy balance, including modifying activity [14], [15] and fine-scale habitat selection [16], [17]. However, thermoregulatory behaviour may be insufficient to totally avoid heat or cold stress [13], it may cause a trade-off with forage availability [17], or individuals may be physiologically constrained to move or select habitats sub-optimally [18]. Furthermore, food intake of endotherms is often inversely related to environmental temperatures [19], [20]. Therefore, despite behavioural responses to the thermal environment, we may still expect non-optimal ambient temperatures to affect the energy balance and productivity of wild populations, just as in domestic livestock production systems [21].

Moose (*Alces alces*) provide a good biological model for investigating temperature mediated habitat-fitness relationships because they are thought to suffer from heat stress during both summer and winter [19]. Indeed, moose typically respond to high ambient temperatures by decreasing activity [22] and increasing their use of thermal shelters such as closed canopy, mature conifer stands [17] although exceptions have been reported [23]. Indirect negative effects of temperature on moose population dynamics have also been observed in both Scandinavia and North America, which are suspected to operate through reduced nutritional quality of forage leading to reduced body condition [24], [25]. Body condition reflects short-term changes in muscle mass and fat reserves, which can be quantified using seasonal mass change analyses. Seasonal mass change is ubiquitous in ungulates [26], [27] and represents an important correlate of individual fitness, especially for females [28]–[31].

In this study, we test whether increased exposure to non-optimal ambient temperatures by individual female moose in southern Norway affects the dynamics of seasonal mass change, which we have shown elsewhere affects the reproductive success of pregnant female moose in this system [31]. To do so we first quantify seasonal thermoregulatory resource selection and activity during summer and winter at the individual level using GPS-collared adult female moose in two populations in southern Norway. Then, rather than attempt to measure the energy balance of individuals in the field, we use seasonal mass change as an index of the resultant energy flows [32]. As such, we evaluate whether individual variation in temperature-mediated resource selection and activity affects seasonal change in body mass.

If ambient temperature is an important factor in the energy balance and, subsequently, body mass dynamics of moose, we would expect the benefits of seeking thermal shelter and being inactive (i.e., optimal thermoregulatory strategy) to outweigh the benefits of foraging at high temperatures (i.e., non-optimal thermoregulatory strategy). We therefore predict that reduced activity and increased selection of thermal cover (e.g., mature conifer forests) during periods of high ambient temperature will be associated with reduced winter mass loss (P_1.1_) and increased summer mass gain (P_1.2_). As a corollary, we predict increased activity and increased selection for forage habitat (e.g., young, successional forest stands) during periods of high ambient temperature to be associated with increased winter mass loss (P_2.1_) and reduced summer mass gain (P_2.2_).

## Methods

### Animal Ethics Statement

All moose were captured, handled and collared by professional wildlife veterinarians using best practice [33], and all efforts were made to minimize suffering. All work was carried out with permission from the national management authority, the Directorate for Nature Management (protocol number: FOTS ID 1428), and evaluated and approved in accordance with the ethical guidelines and legal requirements set by the Norwegian Institute for Nature Research.

### Study Area

Our study areas (Fig. S1) were located in Siljan and Skien municipalities, Telemark county in southern Norway, (59° N, 9°E) and in Stor-Elvdal municipality, Hedmark County, in south-eastern Norway (61° N, 11°E). The vegetation in the two areas was dominated by commercially managed coniferous forest including Norway spruce (*Picea abies*) and Scots pine (*Pinus sylvestris*). Some mixed deciduous stands of birch species (*Betula pubescens* and *B. pendula*), rowan (*Sorbus aucuparia*), willow (*Salix* spp.) and aspen (*Populus tremula*) occurred throughout the areas, particularly in Telemark. Winter moose densities in both areas were estimated to be approximately 1.3 individuals per km^2^ [31], though densities varied locally and were typically higher around feeding stations during winter, especially in Hedmark County. Red deer (*Cervus elaphus*) and roe deer (*Capreolus capreolus*) occurred at much lower densities in both areas. Large predators were essentially absent, with human hunting being the single most important cause of moose mortality.

The climate differed between the study areas, being colder in the more continental Hedmark area, particularly in winter [31]. Average daily minimum and maximum January temperatures during the study period were −2.2°C and 3.1°C respectively in Telemark and −15.5°C and −8.3°C respectively in Hedmark while average daily minimum and maximum July temperatures were 12.2°C and 21.2°C respectively in Telemark and 10.6°C and 20.9°C respectively in Hedmark (Norwegian Meteorological Institute). Snow cover lasted from December to April in Hedmark and a somewhat shorter period in Telemark, with mean February snow depths of 68 cm and 73 cm respectively.

In both the study areas considered here, supplementary feed was provided by local landowners as part of longer-term feeding programmes to reduce traffic accidents. Supplementary feed consists of baled roughage, predominantly mixed graminoids. Feeding stations were located at permanent sites along snow-cleared forest roads with low human activity. The supplementary feed was provided *ad libitum* for 4–6 months of the year (i.e., November through April, with the start and end dependent on annual snow conditions). Our study was carried out in 2007 and 2008 in Telemark and in 2009 and 2010 in Hedmark when an average of 198 t silage/winter and 1538 t/winter respectively was provided.

### Moose Data

Mature adult female moose, each accompanied by a calf, were captured in January 2007–2010 using established techniques [33]. Effort was made to sample adult females from across the spectrum of individual variation in feeding station use (ranging from non-users to heavy users) by capturing individuals at varying distances from feeding stations [31]. Each captured female was fitted with a Global Positioning System (GPS) collar with a Very High Frequency radio transmitter (Tellus Remote GSM, Followit AB, Lindesberg, Sweden), programmed with a 1-h relocation schedule. Bias related to the GPS collars (e.g., location error and fix rate) was low [34].

Body mass was recorded by weighing the restrained moose from a helicopter (mean: 344 kg, range: 235–430 kg, *n = *68). Marked individuals were recaptured and reweighed where possible during March of the same year (mean body mass: 314 kg, range: 228–396 kg, *n* = 56). January and March body mass data were both available for 54 individuals but two were excluded due to GPS collar failure, giving a sample size of 52 individuals for the winter mass change analysis. Blood samples were collected on both capture occasions to determine winter pregnancy status from serum progesterone levels [31]. As such, 47 out of the 52 females (90%) were assumed to be pregnant at the end of winter. In the following autumn, individuals were, where possible, harvested between 16^th^ of September and 23^rd^ of January as part of the annual quota set by the local wildlife board (*n* = 32). This allowed us to age individuals by counting annuli in the cementum of incisor root tips and to collect the ovaries for a separate study. In the first two years, autumn live mass was determined from the sum of the mass of the alimentary and reproductive tracts plus the mass of the whole animal without the alimentary and reproductive tracts (gutless mass). In subsequent years, live mass was estimated from the gutless mass using the relationship live mass = 1.23 * gutless mass +17.36 (R^2^ = 0.83, *n* = 24). The remaining marked individuals that were not shot (n = 16) were recaptured and reweighed by helicopter in December or January. One individual was in extremely poor condition when it was shot, presumed due to illness, so was excluded from analyses. Mean body mass in autumn was 338 kg (range: 224–455 kg, *n* = 47). Presence of a calf or twins in autumn was recorded during both hunting and live capture. If no calf was observed, the female was located again until we were confident of calving status. As such, 21 out of 47 females (45%) had no calf at heel, 23 females (49%) had one calf at heel, and three females (6%) were accompanied by twins in autumn.

### Habitat Maps

Habitat maps were compiled from a combination of digital forest stand maps and satellite land cover maps with a resolution of 50 m × 50 m. In Hedmark, maps of forest stand age and tree species composition were made for the areas of commercially managed forest using satellite data from the Norwegian Forest and Landscape Institute [31]. In Telemark, these satellite data were unavailable for a large part of the study area, so we used ground-truthed commercial forestry maps [34], which accounted for 77% of GPS locations in the area. A satellite data vegetation map produced by the Northern Research Institute was used to classify all remaining areas used by moose in both study areas. Land cover was classified into 6 habitat classes that have previously been shown to influence moose habitat selection in Norway [34]–[36]: mature forest (dense canopy coniferous forest and conifer-dominated stands of felling classes 3–5 of the Norwegian National Forest Inventory), young pine forest (Scots pine stands ≤40 years old, felling classes 1–2), young spruce forest (Norway spruce stands ≤40 years old, felling classes 1–2), deciduous forest (deciduous stands of all ages, including sub-alpine birch woodland), open mixed forest (mixed coniferous or mixed coniferous/deciduous stands ≤40 years old and open canopy mixed or coniferous stands of unknown age) and other (including moorland, heath, bog, agricultural land and open water/ice).

### Temperature, Activity, and Movement Data

Our GPS collars were equipped with a temperature sensor and recorded the temperature during each location attempt. The collars therefore provided local temperature data which are considered more useful than data from weather stations when studying fine-scale behavioural responses of animals to thermal conditions [17]. Collar trials showed that recorded temperatures were closely correlated to ambient temperature as measured by a thermometer (*r_s_* = 0.97) and less closely correlated to conditions recorded by a black globe device (*r_s_* = 0.85) which measured radiant heat load [17]. The GPS collars underestimated the actual radiant heat load experienced by the moose, especially at higher temperatures (Fig. S2), thereby providing a conservative estimate of the subsequent response of moose to thermal conditions. Within each season, moose GPS locations were classified by temperature in relation to seasonal thermoregulation thresholds thought to induce heat stress in moose [19]. Three classes were defined: 1) low ambient temperature (collar temperature <−5°C in winter and <14°C in summer), 2) moderate ambient temperature (≥−5°C <0°C in winter and ≥14°C <20°C in summer) and 3) high ambient temperature (≥0°C in winter and ≥20°C in summer). Although the appropriateness of these thresholds has recently been questioned [23], there is mounting evidence of thermoregulatory behaviour related to these same temperature thresholds in our population [17] and others [22], [37], [38]. We therefore considered them as a suitable starting point to study the effects of individual behavioural responses to thermal conditions on seasonal mass change.

Our GPS collars were equipped with dual axis motion sensors, which record vertical and lateral head and neck movements. During each location attempt the total number of movements (range = 0–92) was stored in the collar memory. We used the movement counts in combination with step length and turning angles between successive GPS locations to distinguish between active and resting locations using *k*-means clustering analysis [39]. First, observations were tallied for each individual into 9 bins for activity, step length, and turning angles. Then, the percentage of observations associated with a bin was calculated for each individual within both seasons (Fig. S3). The clustering procedure classified each GPS observation as either an active or inactive location based on a combination of activity, step length, and turning angle characteristics [39]. Active locations were characterised by relatively high activity counts in combination with relatively short step lengths and sharp turning angles (reflecting foraging behaviour) or by locations with high activity counts in combination with long step lengths and small turning angles (reflecting movement behaviour). In contrast, inactive locations were characterised by relatively low activity counts in combination with relatively short step lengths and sharp turning angles (reflecting resting behaviour). Finally, we calculated the proportion of active fixes in relation to habitat type and temperature class for each individual within a season separately.

### Resource Selection Functions

We estimated seasonal habitat selection patterns for individual moose as a function of temperature class (provided above) and habitat type using resource selection functions (RSFs; [40]). Because ambient temperature directly affects movement of moose at short temporal scales [41], we quantified temperature mediated RSFs at the scale of an individual’s movement trajectory using a matched case-control design [42]. With this approach, each observed (GPS) location (scored 1) is linked to a set of random (available) locations (scored 0), sampled from around the observed location. We associated each observed location with five random locations sampled from around the observed location using the observed step length and turning angle distributions from each individual during a given season (Fig. S3). The individual-based and seasonally-specific RSFs were solved using conditional logistic regression from the R package survival. The selection coefficients (β) estimated by the conditional logistic regression were the log(odds ratio) for a habitat type being selected relative to a reference habitat type (β = 0). In our case, the reference category was set to deciduous forest as most individual moose in the RSF analyses used this forest type in proportion to its availability, which facilitated direct comparison with selection coefficients of the other habitat types included in the analyses.

### Relative Mass Change Analysis

Relative mass change over a season (winter and summer) was modelled using linear regression with individual-specific temperature-dependent resource selection coefficients and habitat- and temperature-dependent proportions of activity as covariates. The response variable was relative mass change, calculated as log(end of season mass/start of season mass) [31]. Due to collinearity between resource selection coefficients and activity estimates of the 3 temperature classes (Variance Inflation Factors >10), we only considered covariates from the low and high ambient temperature classes as we expected the effect on relative mass change to be most pronounced at the extremes of the temperature gradient.

Because of considerable variation among individuals in the date shot or reweighed in winter (March 22^nd^–28^th^) and particularly autumn (September 16^th^–January23^rd^), we included the number of days between seasonal weighing events as a linear continuous covariate in the seasonal mass change models. We found no evidence of a non-linear relationship between the number of days between seasonal weighing events and relative mass change. For the autumn analyses we used the number of days from the beginning of summer (June 1^st^) until autumn weighing or date shot. This covariate was forced into both seasonal mass change models irrespective of its significance (see model selection procedure below). Additional covariates considered in the full models were: pregnancy status (yes or no; winter models only), number of calves at heel (summer models only), year (4 class categorical variable) or study area (2 class categorical variable), autumn status (live or shot; summer models only), and the proportion of time spent at feeding stations (both winter and summer models). Proportion of time spent at feeding stations during each temperature class was calculated for each individual separately as the arcsine square root-transformed proportion of time (i.e., proportion of GPS locations) during winter spent within a 100 m buffer around feeding stations. This buffer size was chosen as it covers the combined distance of the median location error of the GPS collars and the pixel size of our habitat maps. Moreover, it has previously been used to effectively categorize feeding station users and non-users [34]. Because of considerable variation in the distribution among individuals across the altitudinal gradient in both study areas we also considered the mean altitude (m) used during a season as a covariate in the seasonal mass change analyses. Age was not included in the final mass change analyses as preliminary tests revealed no relation between age and seasonal mass change (*r_p_* = −0.132, *P* = 0.667 in winter and *r_p = _*0.068, *P* = 0.816 in summer). This was probably because no yearling females were included (mean age = 7.5 yr ±3.8 SD) and all individuals had calved in the previous year. The Variance Inflation Factor was always <10 between the covariates considered in the full models, confirming weak collinearity among the independent variables.

Model selection was conducted by backward selection with *F* tests using *P* = 0.05 as the threshold for removal of predictor variables [43]. Model comparison between the reduced and the more complicated model was by likelihood ratio tests. To ensure that linear regression models were appropriate we checked homogeneity of the residuals versus the fitted values, normality of the residuals (Shapiro test for normality), and equal variances and independencies among within-group errors. We report the amount of variation explained (R^2^_adj_) for all final models, as well as the partial R^2^ for each covariate separately. Partial R^2^ was used to determine which variables were most influential in relative mass change and was calculated by manually excluding a covariate from the final model and calculating the difference in R^2^_adj_ of the final model and the reduced model [44].

### Thermoregulatory Strategies and between Season Variability

The analyses described above provided insight into the effect of single covariates on seasonal mass change of adult female moose, while controlling for the effect of other covariates. We extended this analysis with the aim of classifying individuals into distinct seasonal thermoregulatory strategies incorporating all influential covariates simultaneously. To do so we employed indirect gradient analysis (i.e., ordination) using principal components analysis (PCA [45]). The PCA ordination method aims to reduce the number of covariates retained in the seasonal mass change analyses to 2 ordination axes in such a way that most of the variation in observed thermoregulatory behaviour is explained. Based on the position in ordination space (the values of PCA ordination axes) individuals with similar thermoregulatory behaviour can be grouped and the effectiveness of the strategy (e.g., optimal, sub-optimal or non-optimal) inferred. PCAs were performed for each season separately, which allowed us to evaluate whether individuals showed variability in their behavioural strategy between seasons. We did not consider the covariate ‘number of days between seasonal weighing events’ in the PCA ordination as this variable does not reflect a thermoregulatory behaviour. Within each season, we tested for differences in relative mass change between behavioural strategies using ANOVA, followed by post hoc paired Tukey HSD tests. We also verified our PCA based thermoregulatory classification with an independent grouping procedure based on hierarchical clustering and *k*-means analysis (see Supporting Information Text S1 for full details).

## Results

### Relative Mass Change

**Figure 1:**
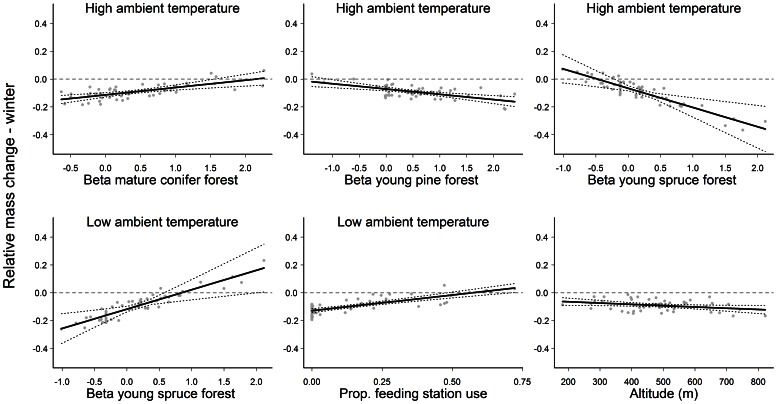
Relative mass change of adult female moose during winter (*n* = 52) in southern Norway as a function of temperature-dependent resource selection coefficients (high ambient temperature was ≥0°C and low ambient temperature was<−5°C) and proportion of feeding station use. Relative mass change over winter was calculated from body mass in January and March (see text). Predictions for each covariate were made while keeping the other variables in the model constant at their mean value. The horizontal dashed line represents no mass change. The dotted lines indicate 95% confidence intervals around the predicted line (solid black line). The grey points are model residuals.

**Figure 2:**
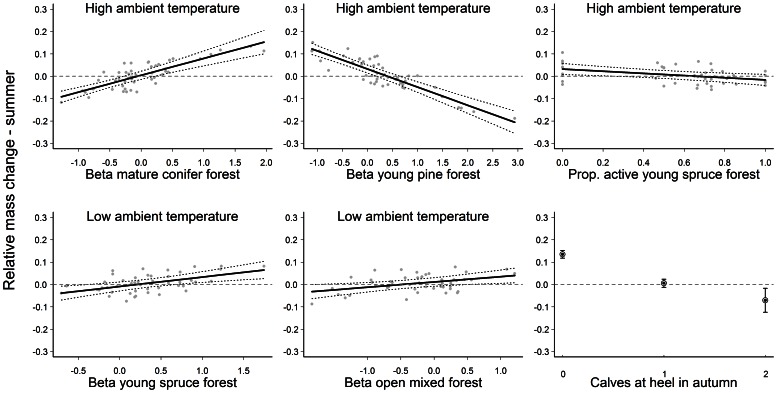
Relative mass change of moose during summer (*n* = 47) in southern Norway as a function of temperature-dependent resource selection coefficients (high ambient temperature was ≥20°C and low ambient temperature was <14°C), proportion of activity, and number of calves at heel in autumn. Relative mass change over summer was calculated from body mass in March and autumn (see text). Predictions for each covariate were made while keeping the other variables in the model constant at their mean value (for calves at heel we used 1 calf). The horizontal dashed line represents no mass change. The dotted lines indicate 95% confidence intervals around the predicted line (solid black line). The grey points are model residuals.

Relative over-winter mass change was influenced most (*F*_1,44_ = 64.63; *P*<0.001; partial R^2^ = 0.349) and positively (β±SE = 0.23±0.028) by the proportional use of feeding stations during periods of low ambient temperature (Fig. 1). Of the 52 GPS collared female moose, 19 did not use feeding stations at low ambient temperatures during winter. Mean (min, max) proportion of time spent at feeding stations of the 33 adult females that did use winter feeding stations at low ambient temperature was 0.26 (0.002, 0.722). Use of winter feeding stations during periods of high ambient temperature did not affect over-winter mass change, and the covariate was not retained in our final model (Table S1). Selection for mature coniferous stands during periods of high ambient temperature was positively related to over-winter mass change (*F*_1,44_ = 15.46; *P*<0.001; partial R^2^ = 0.084; β±SE = 0.053±0.013) whereas selection for young open forest stands during periods of high ambient temperature was negatively related to over-winter mass change (pine: *F*_1,44_ = 17.53; *P*<0.001; partial R^2^ = 0.095; β±SE = −0.038±0.009 and spruce: *F*_1,44_ = 10.39; *P* = 0.002; partial R^2^ = 0.056; β±SE = −0.138±0.043). In contrast, selection of young spruce forest during periods of low ambient temperature was positively related to over-winter mass change (*F*_1,44_ = 9.42; *P* = 0.004; partial R^2^ = 0.051; β±SE = 0.139±0.045). Mean altitude (m) used during winter was negatively related to over-winter mass change (*F*_1,44_ = 4.61; *P* = 0.037; partial R^2^ = 0.025; β±SE = −0.0001±0.00004). The number of days between winter weighing events (Jan-Mar) was negatively correlated with over-winter mass change (β±SE = −0.002±0.001) though the effect was not significant and did not explain much variation in the data (*F*_1,44_ = 1.24; *P* = 0.271; partial R^2^ = 0.007). Activity during winter did not appear in our final over-winter mass change model. The final model accounted for 72% of the observed variation in the data. Relative mass change during summer was influenced most by the observed number of calves at heel in autumn (*F*_2,38_ = 75.36; *P*<0.001; partial R^2^ = 0.349; Table S2). Female moose accompanied by twins lost mass over summer, while females accompanied by singletons showed little mass change and females without calves gained mass (Fig. 2). Selection for mature coniferous stands under high ambient temperatures was positively related to over-summer mass gain (*F*_1,38_ = 47.70; *P*<0.001; partial R^2^ = 0.141; β±SE = 0.082±0.011) whereas selection for young pine stands and proportion of activity in young spruce stands during high ambient temperatures were negatively related to over-summer mass gain (pine: *F*_1,38_ = 97.75; *P*<0.001; partial R^2^ = 0.141; β±SE = −0.07±0.009, and spruce: *F*_1,38_ = 7.78; *P* = 0.008; partial R^2^ = 0.017; β±SE = −0.05±0.020). In addition, at low ambient temperatures, selection for both open mixed forest and young spruce forest were positively related to over-summer mass gain (open mixed: *F*_1, 38_ = 6.95; *P* = 0.012; partial R^2^ = 0.017; β±SE = 0.024±0.009, and young spruce: *F*_1,38_ = 11.71; *P* = 0.002; partial R^2^ = 0.021; β±SE = 0.036±0.013). The number of days between 1st June and autumn weighing was positively correlated with over-summer mass change (β±SE = 0.002±0.002) though the effect was not significant and did not explain much variation in the data (*F*_1,38_ = 0.364; *P* = 0.549; partial R^2^ = 0.001).The final summer model accounted for 85% of the variation in the data.

### Thermoregulatory Strategies and between Season Variability

**Figure 3:**
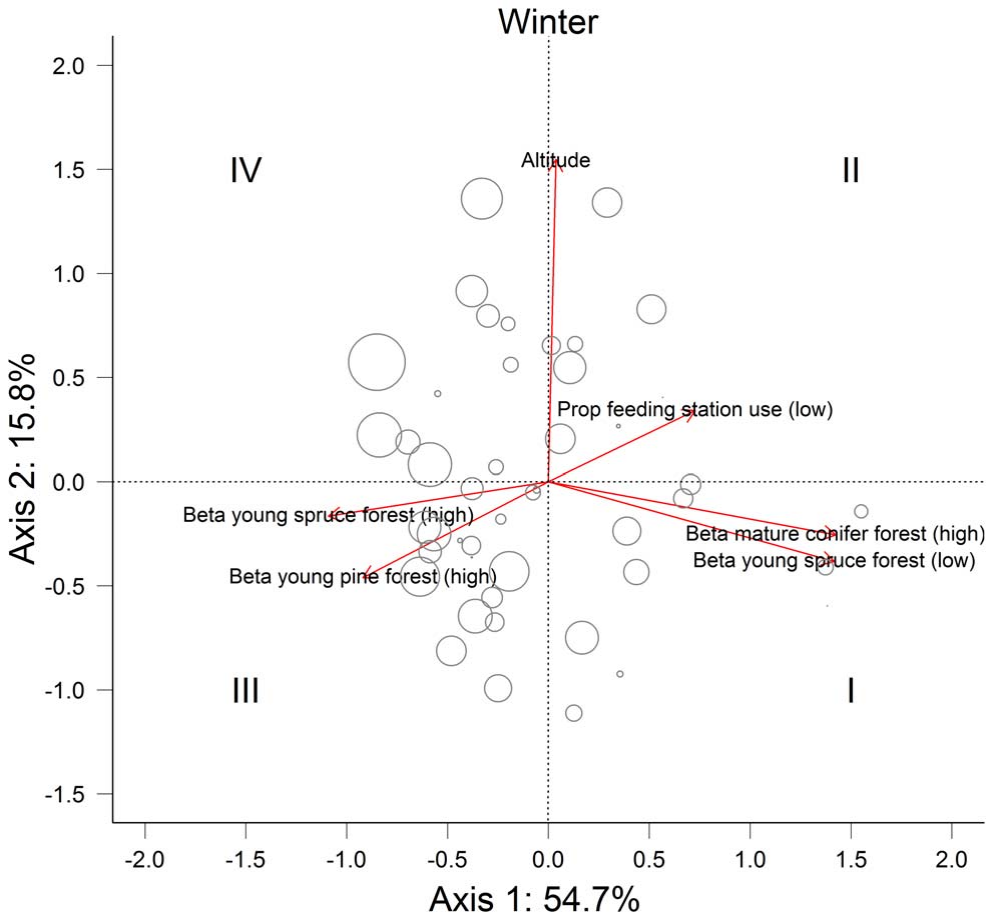
PCA ordination biplot on the covariates influencing over winter mass change for adult female moose (*n* = 52) in southern Norway. Roman numerals indicate the four quarters of the ordination biplot and represent different thermoregulatory strategies ranging from optimal (I) to non-optimal (IV). Circles represent individual moose plotted relative to their scores of the PCA axes and circle size is proportional to over winter mass change (i.e., the larger the circle the more mass was lost).

**Figure 4:**
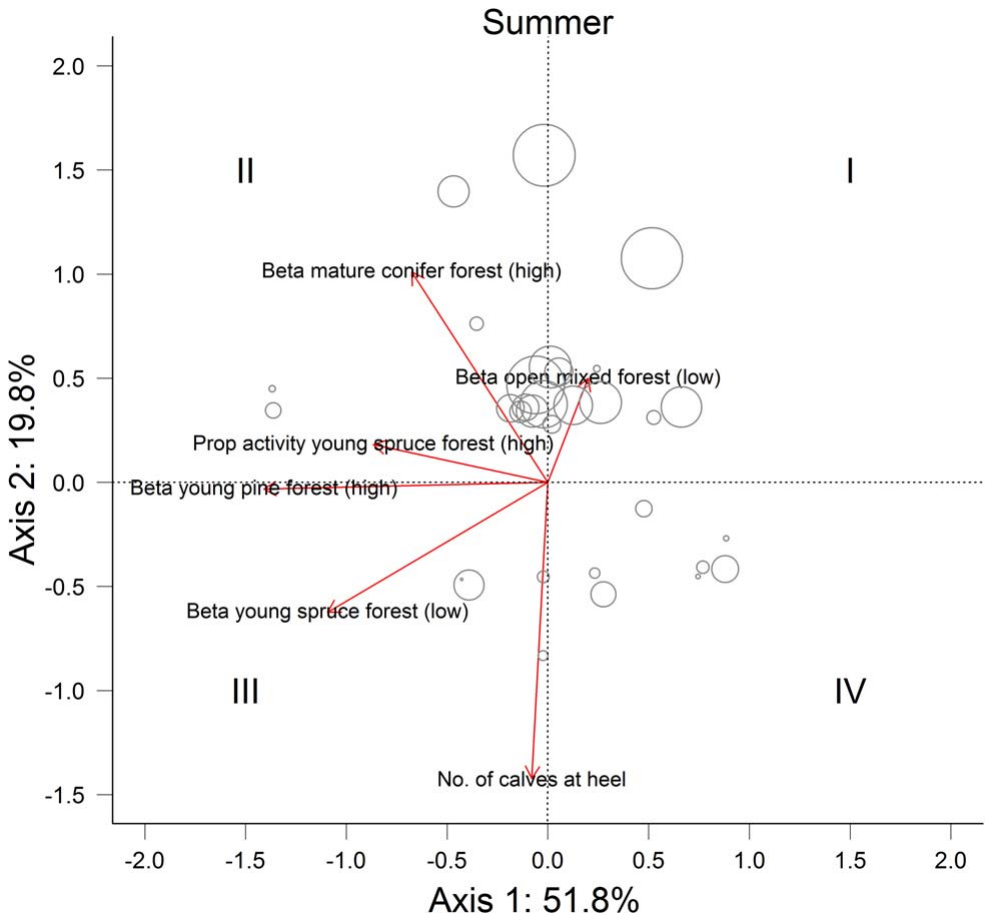
PCA ordination biplot on the covariates influencing over summer mass change for adult female moose (*n* = 47) in southern Norway. Roman numerals indicate the four quarters of the ordination biplot and represent different thermoregulatory strategies ranging from optimal (I) to non-optimal (IV). Circles represent individual moose plotted relative to their scores of the PCA axes and circle size is proportional to over summer mass change (i.e., the larger the circle the more mass was gained).

PCA ordination of the behavioural covariates influencing seasonal mass change revealed clear patterns in both seasons (Figs. 3 and 4). For the over winter analyses, the first PCA axis explained a substantial proportion of the between individual variation in behaviour (54.7%) and was positively related to selection for mature conifer forest during high ambient temperatures (eigenvalue = 1.425), selection for young spruce forest at low ambient temperature (eigenvalue = 1.417) and negatively related to selection for young spruce forest at high ambient temperature (eigenvalue = −1.09). Feeding station use was also positively related to the first PCA axis (eigenvalue = 0.727). As such, individuals with a positive score on the first PCA axis displayed a more optimal thermoregulatory strategy and/or made more use of feeding stations than individuals positioned at the opposite end of PCA axis 1. The second PCA axis explained 15.8% of the variation and primarily partitioned individual behaviour on seasonal use of altitude (eigenvalue = 1.547). Individuals with a positive score on the second PCA axis used higher areas than individuals with a negative value on the second PCA axis.

During summer, the first PCA axis explained 51.8% of the behavioural variation and was influenced by selection for young pine forest during high ambient temperatures (eigenvalue = −1.409) and proportion of activity in young spruce forest at high ambient temperature (eigenvalue = −1.093). As such, individuals with a positive score on the first PCA axis displayed a more optimal thermoregulatory strategy compared to individuals with a negative score. The second PCA axis explained 19.8% of the variation and partitioned individual behaviour based on the number of calves at heel in autumn (eigenvalue = −1.422), selection for mature conifer forest at high ambient temperature (eigenvalue = 1.011) and selection for open mixed forest at low ambient temperature (eigenvalue = 0.924). Individuals with a positive score on the second PCA axis had a more optimal thermoregulatory strategy compared to individuals with a negative score.

**Figure 5:**
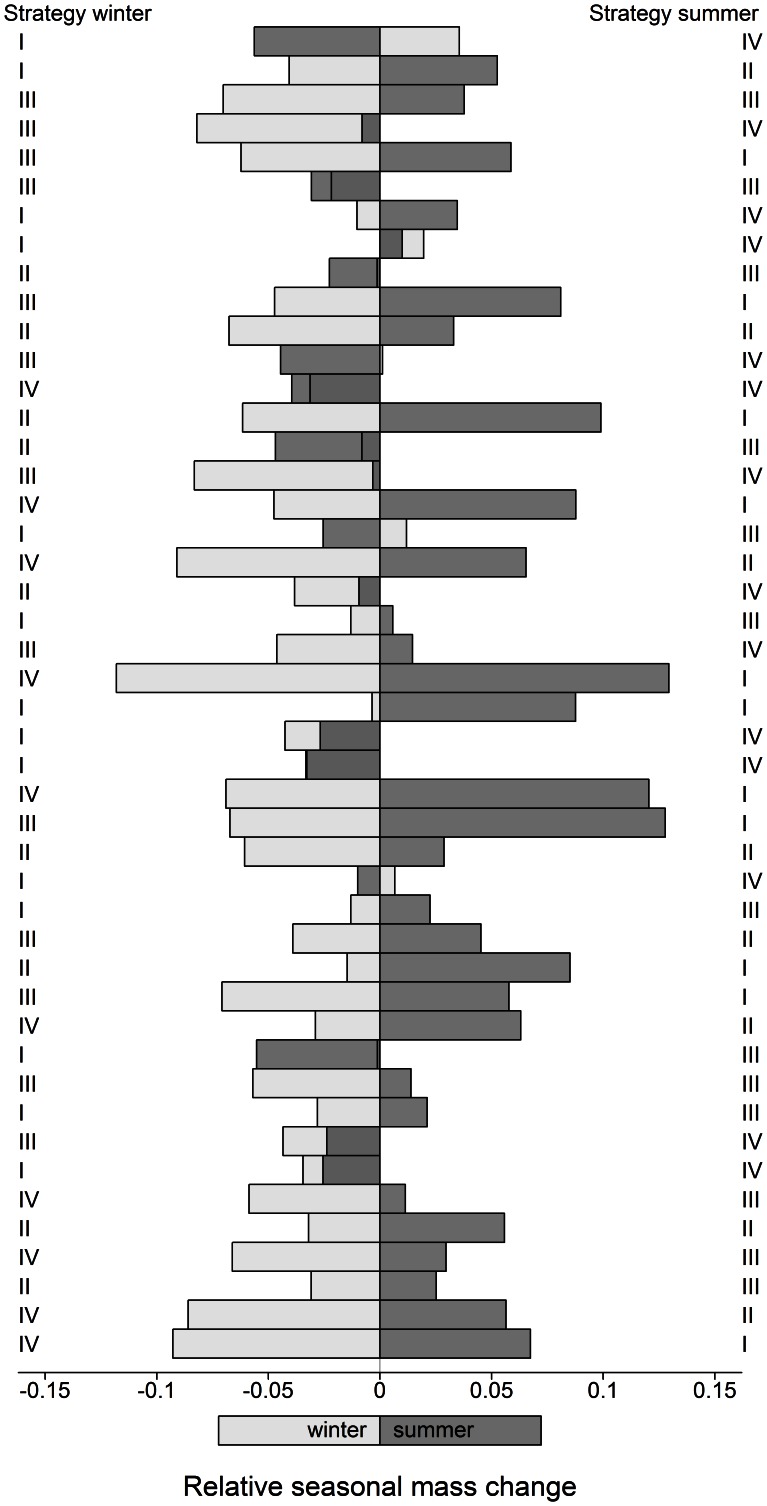
Plot showing the seasonal thermoregulatory strategy and relative mass change for each adult female moose in southern Norway. Only individuals for which mass change over both seasons was known are included shown (*n* = 46).

The first two PCA axes clearly represented variation in thermoregulatory behaviour within both seasons. We therefore used them to operationally define 4 thermoregulatory strategies associated with the four quarters of the seasonal PCA ordination biplot (strategies I–IV; Figs. 3 and 4). Hierarchical clustering and *k*-means analysis on the same input data corroborated a four-cluster grouping of individual behaviour (Figs. S4 and S5). Relative mass change differed between the four behavioural strategies in both seasons (winter: *F*_3,42_ = 12.25; *P*<0.001, summer: *F*_3,42_ = 43.37; *P*<0.001 ). As expected, individuals using an optimal thermoregulatory strategy (strategy I) lost less mass over winter or gained more mass over summer (Fig. 5) than individuals using a sub-optimal (III) or non-optimal (IV) strategy (Tukey HSD: *P*<0.001 for both seasons and cross-comparisons). During winter, we found no differences in relative mass change between strategy I and II (Tukey HSD: *P* = 0.12). We observed substantial variability in thermoregulatory strategies employed by individuals between seasons (Fig. 5). For example, 15 individuals employed a non-optimal (IV) or sub-optimal (III) thermoregulatory strategy during winter but an optimal (I) or better sub-optimal (II) strategy during summer. In contrast, 12 individuals employed an optimal (I) or sub-optimal (II) strategy in winter, but behaved sub-optimal (III) or non-optimal (IV) in summer. Only 6 individuals consistently used an optimal (I) or sub-optimal (II) thermoregulatory strategy in both seasons, while 13 individuals consistently used a sub-optimal (III) or non-optimal (IV) thermoregulatory strategy in both seasons.

## Discussion

The behavioural response of both endotherms and ectotherms to thermal conditions has become a topic of growing interest due to current and predicted global warming [46]. Simultaneously, the fitness consequences of an individual’s habitat choice (i.e., habitat-fitness [1] or habitat-performance relationships [3]) are increasingly being uncovered using fitness indices such as body mass, reproduction, and survival [1], [2]. To the best of our knowledge, ours is the first study to link the two for a free-ranging, endothermic species.

We show that behavioural responses to ambient temperature have the potential to impact on the body condition of adult female moose, a heat-sensitive species. Individual variation in resource selection and activity affected mass change in both seasons. As such, and in line with our predictions (P_1.1_ and P_1.2_), individuals that selected for mature coniferous forest under thermally stressful conditions or young successional forest stands, abundant in forage [35], at low ambient temperatures (i.e., optimal thermoregulatory strategy) lost less mass during winter and gained more mass over summer. Contrastingly, relative mass change in both seasons was negatively affected when individuals selected for young successional forest stands under thermally stressful conditions (i.e., non-optimal thermoregulatory strategy and as expected by P_2.1_ and P_2.2_). Therefore, the most effective strategy for adult female moose to cope with thermally stressful conditions is to adopt a time-minimizer foraging strategy (i.e., minimizing the time spent in foraging activities to fulfil minimum energetic requirement [47]) rather than to adopt an energy-maximizer strategy, a behavioural response also suggested to be employed by other large herbivores [14], [15].

Direct effects of temperature on behaviour and seasonal mass change, as shown here, may have important implications for population demography and dynamics [9], [48]. Indeed, seasonal mass change was an important driver of reproductive success and failure in both the moose populations studied here, with over-winter mass loss affecting spring calving success and over-summer calf survival [31]. Furthermore over-summer mass gain can influence ovulation and pregnancy rates in the subsequent reproductive cycle [30], [49]. The seasonal temperatures observed during our study in Telemark were similar to the 30-year mean and variance [41]. Thus our results are not due to extreme climatic events, which are well-known to influence the performance of large herbivores [9], [11], [50].

Seasonal mass change in herbivores is ubiquitous, resulting from seasonal changes in forage quality and availability [26]. Pelletier et al. [27], showed that relative seasonal mass change of bighorn sheep (*Ovis canadensis*) is also influenced by substantial genetic variation and may be heritable. Their study neatly highlights a potential evolutionary response to natural selection in seasonal mass change. Although we lack the data to test whether seasonal changes in body mass of moose are currently under natural selection to cope with high ambient temperatures (i.e., climate change), it seems a plausible hypothesis that deserves further attention. Another mechanism that can explain population responses to climate change is phenotypic plasticity [51]. We found that some moose showed non-optimal thermoregulatory strategies in both seasons suggesting that some individuals may be unable to respond adaptively (i.e., low plasticity) to increases in ambient temperature. Our findings therefore substantiate previous suggestions that current ambient temperatures may be a contributing factor to the decline in demographic performance of moose populations living in southern Norway [24], [31] and North America [25]. Indeed, behavioural strategies employed above critical temperature thresholds may have important eco-evolutionary consequences [52], as also indicated by recent observations that morphology (ear, rostrum, and leg length) of moose is related to climatic conditions observed during summer [53].

Temperature-dependent habitat selection had more influence on seasonal mass change than individual activity (Figs. 1 and 2). The only effect of activity that we found was a negative one within young spruce stands (foraging habitat) at high ambient temperatures in summer (as expected by P_2.2_, Fig. 2). This result, as well as the lack of an effect of feeding station use at high temperatures on over-winter mass change, corroborates our previous suggestion that an energy maximizing strategy during thermally stressful conditions is disadvantageous in terms of seasonal mass change. During winter, activity did not appear in our final model of mass change. This may partly be explained by the fact that large herbivores reduce activity more during winter compared to summer [54], typically related to environmental constraints on locomotion and reduced diet quality [55]. Indeed, moose are often faced with low quality, high fibre forage during winter, leading to an increase in the proportion of time spent ruminating [32]. It is likely that rumination sets similar physiological constraints on activity across individuals, leading to little individual variation [56].

The most influential variable in our final summer mass change model was the number of calves at heel in autumn. As lactation greatly increases energy expenditure, affecting maternal body growth and fecundity in moose [57] as well as other mammals [49], [58], it was unsurprising that barren females gained the most mass over summer (Fig. 2). During winter, use of supplementary feeding stations at low ambient temperatures was the most influential factor affecting mass change. Indeed, improving over-winter body mass and condition is the primary goal of many winter feeding programmes [59].

Individual variation in movement, activity, and habitat or resource selection strategies is common in many species, including moose [60], and may hold important ecological information about the underlying gradient(s) that influence animal fitness [3]. Indeed, we found considerable individual variation in temperature-dependent RSF coefficients, and to a lesser extent activity, with a clear gradient in thermoregulatory strategies. While some individuals selected optimally for thermal cover when temperatures were high or for foraging habitat under low ambient temperatures, others behaved in an apparently sub-, or non-optimal manner. There are a number of possible explanations for the latter. For example, this behaviour may be linked to individual physiological condition [18], a longer-term energy maximisation strategy [30] or individual differences in thermal tolerance [13]. Alternatively environmental stochasticity or incomplete knowledge of the ecological landscape may lead to sub-optimal behaviour [18]. Free-ranging ungulates must balance a number of limiting, potentially conflicting ecological factors so, from a life-history perspective, they should employ strategies that minimise the maximum detriment to fitness [30] rather than optimise short-term behaviour. We could therefore expect individuals to adopt a suite of temperature-dependent behaviours and habitat choices which together maximise the energy balance under given environmental conditions, even though this suite may include behaviours which alone appear sub-optimal. Clearly a better understanding of the mechanisms that drive the sort of climate-related behavioural-fitness effects reported here, is an important prerequisite for appropriate conservation and wildlife management [46], [48]. The influence of climate on animal behaviour and, subsequently, fitness is expected to intensify as global warming continues.

## Supporting Information

Figure S1 **Study areas in southern Norway.**
(DOC)

Figure S2 **Correlation between simultaneous temperature recordings by GPS collars, thermometers, and a black globe device.**
(DOC)

Figure S3 **Seasonal movement characteristics of adult female moose in southern Norway.**
(DOC)

Figure S4 **Results of hierarchical cluster and** ***k*** **-means analyses to quantify thermoregulatory strategies by adult female moose during winter.**
(DOC)

Figure S5 **Results of hierarchical cluster and** ***k*** **-means analyses to quantify thermoregulatory strategies by adult female moose during summer.**
(DOC)

Table S1 **Covariates influencing over-winter mass change for adult female moose in southern Norway.**
(DOC)

Table S2 **Covariates influencing over-summer mass change for adult female moose in southern Norway.**
(DOC)

Text S1 **Evaluating the PCA method with cluster analyses to quantify thermoregulatory strategies.**
(DOC)

## References

[1] McLoughlin PD, Boyce MS, Coulson T, Clutton-Brock T (2006) Lifetime reproductive success and density-dependent, multi-variable resource selection. Proc R Soc B-Biol Sci 273: 1449–1454 doi:10.1098/rspb.2006.3486 10.1098/rspb.2006.3486 PMC1560319 16777736

[2] Van Moorter B, Gaillard JM, McLoughlin PD, Delorme D, Klein F, et al (2009) Maternal and individual effects in selection of bed sites and their consequences for fawn survival at different spatial scales. Oecologia 159: 669–678 doi:10.1007/s00442-008-1245-1 19089457 10.1007/s00442-008-1245-1

[3] Gaillard J-M, Hebblewhite M, Loison A, Fuller M, Powell R, et al (2010) Habitat-performance relationships: finding the right metric at a given spatial scale. Phil Trans R Soc B 365: 2255–2265 doi:10.1098/rstb.2010.0085 20566502 10.1098/rstb.2010.0085 PMC2894964

[4] Nielsen SE, Herrero S, Boyce MS, Mace RD, Benn B, et al (2004) Modelling the spatial distribution of human-caused grizzly bear mortalities in the Central Rockies ecosystem of Canada. Biol Conserv 120: 101–113 doi:10.1016/j.biocon.2004.02.020

[5] Creel S, Christianson D (2008) Relationships between direct predation and risk effects. Trends Ecol Evol 23: 194–201 doi:10.1016/j.tree.2007.12.004 18308423 10.1016/j.tree.2007.12.004

[6] Stien A, Loe LE, Mysterud A, Severinsen T, Kohler J, et al (2010) Icing events trigger range displacement in a high-arctic ungulate. Ecology 91: 915–920. 20426348 10.1890/09-0056.1

[7] Parmesan C (2006) Ecological and evolutionary responses to recent climate change. Annu Rev Ecol Syst 37: 637–669 doi:10.1146/annurev.ecolsys.37.091305.110100

[8] Post E, Brodie J, Hebblewhite M, Anders AD, Maier JAK, et al (2009) Global population dynamics and hot spots of response to climate change. Bioscience 59: 489–497 doi:10.1525/bio.2009.59.6.7

[9] Hansen BB, Aanes R, Herfindal I, Kohler J, Sæther B-E (2011) Climate, icing, and wild arctic reindeer: past relationships and future prospects. Ecology 92: 1917–1923. 22073783 10.1890/11-0095.1

[10] Avery R (1978) Activity patterns, thermoregulation and food-consumption in 2 sympatric lizard species (*Podarcis-muralis* and *P-sicula*) from central Italy. J Anim Ecol 47: 143–158 doi:10.2307/3928

[11] Garel M, Loison A, Gaillard J, Cugnasse J, Maillard D (2004) The effects of a severe drought on mouflon lamb survival. Proc R Soc Lond Ser B-Biol Sci 271: S471–S473 doi:10.1098/rsbl.2004.0219 10.1098/rsbl.2004.0219 PMC1810106 15801607

[12] Mysterud A, Yoccoz NG, Langvatn R, Pettorelli N, Stenseth NC (2008) Hierarchical path analysis of deer responses to direct and indirect effects of climate in northern forest. Philos Trans R Soc B-Biol Sci 363: 2359–2368 doi:10.1098/rstb.2007.2206 10.1098/rstb.2007.2206 PMC2606786 18006411

[13] Boyles JG, Seebacher F, Smit B, McKechnie AE (2011) Adaptive thermoregulation in endotherms may alter responses to climate change. Integr Comp Biol 51: 676–690. 21690108 10.1093/icb/icr053

[14] Aublet JF, Festa-Bianchet M, Bergero D, Bassano B (2009) Temperature constraints on foraging behaviour of male Alpine ibex (*Capra ibex*) in summer. Oecologia 159: 237–247 doi:10.1007/s00442-008-1198-4 18987895 10.1007/s00442-008-1198-4

[15] Bourgoin G, Garel M, Blanchard P, Dubray D, Maillard D, et al (2011) Daily responses of mouflon (*Ovis gmelini musimon* x Ovis sp.) activity to summer climatic conditions. Can J Zool-Rev Can Zool 89: 765–773 doi:10.1139/Z11-046

[16] Bowyer RT, Kie JG (2009) Thermal landscapes and resource selection by black-tailed deer: Implications for large herbivores. Calif Fish Game 95: 128–139.

[17] van Beest FM, Van Moorter B, Milner JM (2012) Temperature-mediated habitat use and selection by a heat-sensitive northern ungulate. Anim Behav 84: 723–735 doi:10.1016/j.anbehav.2012.06.032

[18] Galanthay TE, Flaxman SM (2012) Generalized movement strategies for constrained consumers: ignoring fitness can be adaptive. Am Nat 179: 475–489 doi:10.1086/664625 22437177 10.1086/664625

[19] Renecker LA, Hudson RJ (1986) Seasonal energy expenditures and thermoregulatory responses of moose. Can J Zool 64: 322–327.

[20] Huey RB (1991) Physiological consequences of habitat selection. Am Nat 137: S91–S115.

[21] Burthe S, Butler A, Searle KR, Hall SJG, Thackeray SJ, et al (2011) Demographic consequences of increased winter births in a large aseasonally breeding mammal (*Bos taurus*) in response to climate change. J Anim Ecol 80: 1134–1144 doi:10.1111/j.1365-2656.2011.01865.x 21668894 10.1111/j.1365-2656.2011.01865.x

[22] Dussault C, Ouellet JP, Courtois R, Huot J, Breton L, et al (2004) Behavioural responses of moose to thermal conditions in the boreal forest. Ecoscience 11: 321–328.

[23] Lowe SJ, Patterson BR, Schaefer JA (2010) Lack of behavioral responses of moose (*Alces alces*) to high ambient temperatures near the southern periphery of their range. Can J Zool 88: 1032–1041 doi:10.1139/z10-071

[24] Grøtan V, Sæther BE, Lillegard M, Solberg EJ, Engen S (2009) Geographical variation in the influence of density dependence and climate on the recruitment of Norwegian moose. Oecologia 161: 685–695 doi:10.1007/s00442-009-1419-5 19657678 10.1007/s00442-009-1419-5

[25] Lenarz MS, Nelson ME, Schrage MW, Edwards AJ (2009) Temperature mediated moose survival in northeastern Minnesota. J Wildl Manage 73: 503–510 doi:10.2193/2008-265

[26] Festa-Bianchet M, King WJ, Jorgenson JT, Smith KG, Wishart WD (1996) The development of sexual dimorphism: seasonal and lifetime mass changes in bighorn sheep. Can J Zool 74: 330–342 doi:10.1139/z96-041

[27] Pelletier F, Réale D, Garant D, Coltman DW, Festa-Bianchet M, et al (2007) Selection on heritable seasonal phenotypic plasticity of body mass. Evolution 61: 1969–1979 doi:10.1111/j.1558-5646.2007.00160.x 17683438 10.1111/j.1558-5646.2007.00160.x

[28] Clutton-Brock TH, Stevenson IR, Marrow P, MacColl AD, Houston AI, et al (1996) Population fluctuations, reproductive costs and life-history tactics in female Soay sheep. J Anim Ecol 65: 675–689.

[29] Festa-Bianchet M, Gaillard JM, Jorgenson JT (1998) Mass- and density-dependent reproductive success and reproductive costs in a capital breeder. Am Nat 152: 367–379 doi:10.1086/286175 18811445 10.1086/286175

[30] Parker KL, Barboza PS, Gillingham MP (2009) Nutrition integrates environmental responses of ungulates. Funct Ecol 23: 57–69 doi:10.1111/j.1365-2435.2009.01528.x

[31] Milner JM, van Beest FM, Solberg EJ, Storaas T (2013) Reproductive success and failure – the role of winter body mass in reproductive allocation in Norwegian moose. Oecologia: in press. doi:10.1007/s00442-012-2547-x 10.1007/s00442-012-2547-x 23223863

[32] Moen R, Pastor J, Cohen Y (1997) A spatially explicit model of moose foraging and energetics. Ecology 78: 505–521.

[33] Arnemo JM, Kreeger TJ, Soveri T (2003) Chemical immobilization of free-ranging moose. Alces 39: 243–253.

[34] van Beest FM, Loe LE, Mysterud A, Milner JM (2010) Comparative space use and habitat selection of moose around feeding stations. J Wildl Manage 74: 219–227.

[35] van Beest FM, Mysterud A, Loe LE, Milner JM (2010) Forage quantity, quality and depletion as scale-dependent mechanisms driving habitat selection of a large browsing herbivore. J Anim Ecol 79: 910–922 doi:10.1111/j.1365-2656.2010.01701.x 20443990 10.1111/j.1365-2656.2010.01701.x

[36] Herfindal I, Tremblay JP, Hansen BB, Solberg EJ, Heim M, et al (2009) Scale dependency and functional response in moose habitat selection. Ecography 32: 849–859 doi:10.1111/j.1600-0587.2009.05783.x

[37] Schwab FE, Pitt MD (1991) Moose selection of canopy cover types related to operative temperature, forage, and snow depth. Can J Zool 69: 3071–3077.

[38] Renecker LA, Hudson RJ (1990) Behavioral and thermoregulatory of moose to high ambient temperatures and insect harassment in Aspen-dominated forests. Alces 26: 66–72.

[39] Van Moorter B, Visscher DR, Jerde CL, Frair JL, Merrill EH (2010) Identifying movement states from location data using cluster analysis. J Wildl Manage 74: 588–594.

[40] Manly BFJ, McDonald LL, Thomas DL, McDonald TL, Erickson WP (2002) Resource selection by animals: Statistical analysis and design for field studies. Dordrecht, The Netherlands: Kluwer Academic Publishers. 240 p.

[41] van Beest FM, Rivrud IM, Loe LE, Milner JM, Mysterud A (2011) What determines variation in home range size across spatiotemporal scales in a large browsing herbivore? J Anim Ecol 80: 771–785. 21388373 10.1111/j.1365-2656.2011.01829.x

[42] Boyce MS (2006) Scale for resource selection functions. Divers Distrib 12: 269–276 doi:10.1111/j.1366-9516.2006.00243.x

[43] Murtaugh PA (2009) Performance of several variable-selection methods applied to real ecological data. Ecol Lett 12: 1061–1068 doi:10.1111/j.1461-0248.2009.01361.x 19702634 10.1111/j.1461-0248.2009.01361.x

[44] Edwards LJ, Muller KE, Wolfinger RD, Qaqish BF, Schabenberger O (2008) An Rˆ2 statistic for fixed effects in the linear mixed model. Statistics in Medicine 27: 6137–6157 doi:10.1002/sim.3429 18816511 10.1002/sim.3429 PMC2587505

[45] Bertolo A, Pepino M, Adams J, Magnan P (2011) Behavioural Thermoregulatory Tactics in Lacustrine Brook Charr, *Salvelinus fontinalis* . PLoS One 6: e18603 doi:10.1371/journal.pone.0018603 21490935 10.1371/journal.pone.0018603 PMC3072417

[46] Knowlton JL, Graham CH (2010) Using behavioral landscape ecology to predict species’ responses to land-use and climate change. Biol Conserv 143: 1342–1354 doi:10.1016/j.biocon.2010.03.011

[47] Schoener TW (1971) Theory of feeding strategies. A Rev Ecol Syst 2: 369–404.

[48] Mysterud A, Sæther BE (2011) Climate change and implications for the future distribution and management of ungulates in Europe. In: Putman R, Andersen R, Apollonio M, editors. Ungulate management in Europe; problems and practices. Cambridge, UK.: Cambridge University Press. 349–375.

[49] Festa-Bianchet M (1998) Condition-dependent reproductive success in bighorn ewes. Ecol Lett 1: 91–94.

[50] Rughetti M, Festa-Bianchet M (2012) Effects of spring–summer temperature on body mass of chamois. J Mammal 93: 1301–1307 doi:10.1644/11-MAMM-A-402.1

[51] Charmantier A, McCleery RH, Cole LR, Perrins C, Kruuk LEB, et al (2008) Adaptive phenotypic plasticity in response to climate change in a wild bird population. Science 320: 800–803 doi:10.1126/science.1157174 18467590 10.1126/science.1157174

[52] Henry RC, Bocedi G, Travis JMJ (2013) Eco-evolutionary dynamics of range shifts: elastic margins and critical thresholds. J Theor Biol 321. doi:10.1016/j.jtbi.2012.12.004 10.1016/j.jtbi.2012.12.004 23246816

[53] Lundmark C (2008) Morphological and behavioural adaptations of moose to climate, snow, and forage [PhD-thesis]. Umea: Swedish University of Agricultural Sciences, Sweden.

[54] Schmitz OJ (1991) Thermal constraints and optimization of winter feeding and habitat choice in white-tailed deer. Holarctic Ecol 14: 104–111.

[55] Mysterud A, Østbye E (1999) Cover as a habitat element for temperate ungulates: Effects on habitat selection and demography. Wildl Soc Bull 27: 385–394.

[56] Risenhoover KL (1986) Winter activity patterns of moose in interior Alaska. J Wildl Manage 50: 727–734 doi:10.2307/3800990

[57] Sand H (1998) Costs of reproduction in female moose (*Alces alces*) as measured by means of phenotypic correlations. Can J Zool 76: 187–193.

[58] Clutton-Brock TH, Albon SD, Guinness FE (1989) Fitness costs of gestation and lactation in wild mammals. Nature 337: 260–262. 2911365 10.1038/337260a0

[59] Putman RJ, Staines BW (2004) Supplementary winter feeding of wild red deer *Cervus elaphus* in Europe and North America: justifications, feeding practice and effectiveness. Mammal Rev 34: 285–306.

[60] Leblond M, Dussault C, Ouellet JP (2010) What drives fine-scale movements of large herbivores? A case study using moose. Ecography 33: 1102–1112 doi:10.1111/j.1600-0587.2009.06104.x

