# Supplementary material for: Behavioural Responses to Thermal Conditions Affect Seasonal Mass Change in a Heat-Sensitive Northern Ungulate
Source: PLoS One. 2013 Jun 11;8(6):e65972. doi: 10.1371/journal.pone.0065972 (PMC3679019; doi:10.1371/journal.pone.0065972)
Supplement: Figure S1 — Study areas in southern Norway. (DOC) [file pone.0065972.s001.doc]

**Supporting Information**

Behavioural responses to thermal conditions affect seasonal mass change in a heat-sensitive northern ungulate – van Beest & Milner

**Figure S1.**

Geographical position and altitude (m) of the two study areas in southern Norway; Telemark area (A) and Hedmark area (B). Euclidean distance between the centres of the two areas is ca. 250 km. Supplementary feeding stations present in the two areas at the time of this study are indicated in red.
