# Supplementary material for: Behavioural Responses to Thermal Conditions Affect Seasonal Mass Change in a Heat-Sensitive Northern Ungulate
Source: PLoS One. 2013 Jun 11;8(6):e65972. doi: 10.1371/journal.pone.0065972 (PMC3679019; doi:10.1371/journal.pone.0065972)

**Supporting Information**

Behavioural responses to thermal conditions affect seasonal mass change in a heat-sensitive northern ungulate – van Beest & Milner

**Figure S2**

Plot showing the correlation between simultaneous temperature (°C) recordings (*n* = 1698 in summer, *n* = 69 in winter) by GPS collars and thermometers (panels on left) and GPS collars and a black globe device (panels on right), which measures radiant heat load. The red dashed line shows the 1:1 relationship. In both seasons the GPS collars deviated little from ambient temperature as measured by temperature loggers. In contrast, the GPS collars underestimated radiant heat load (measured by black globe device) especially at higher temperatures in both seasons, thereby providing a conservative estimate of the subsequent response of moose to thermal conditions.


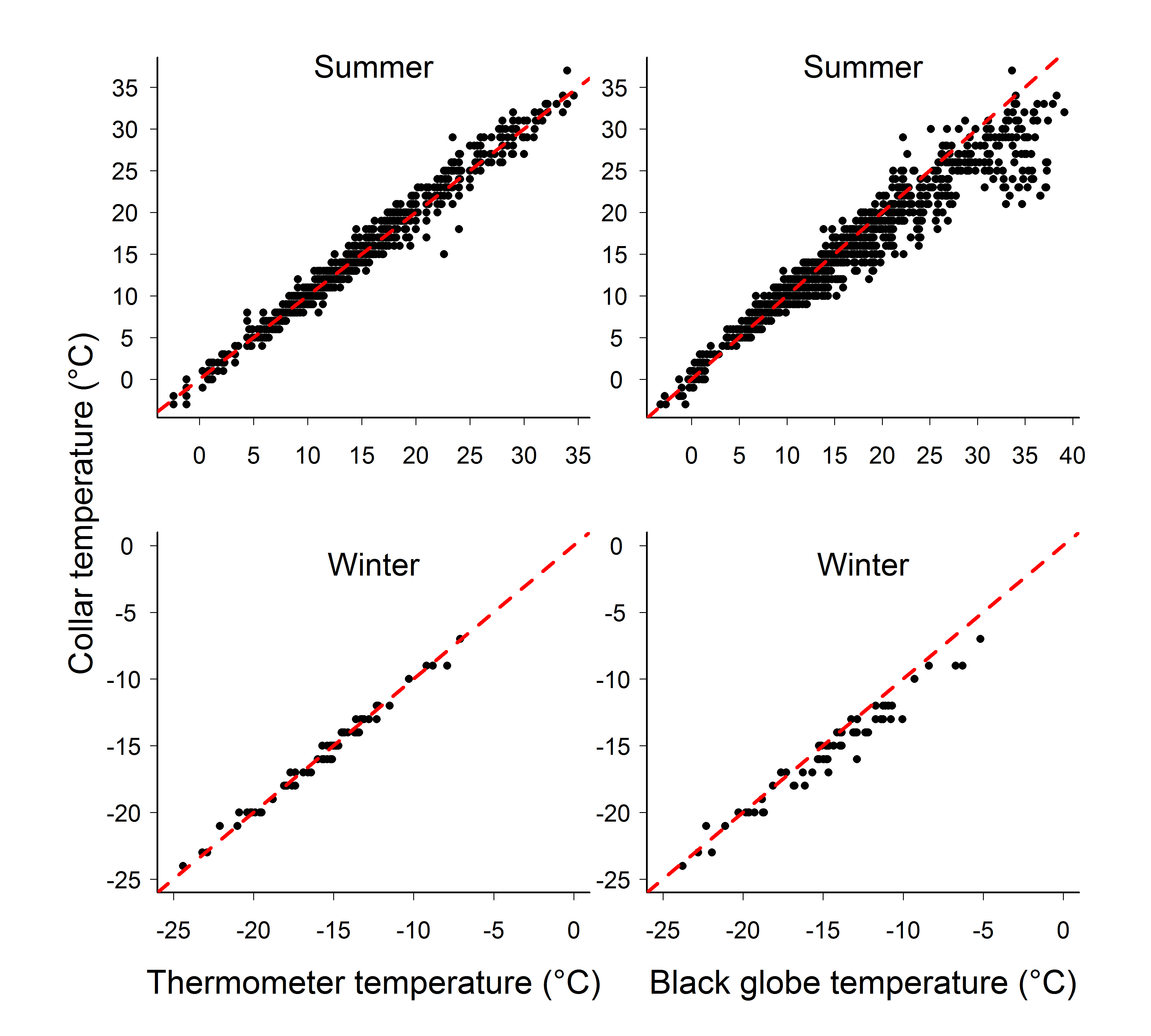

Supplement: Figure S2 — Correlation between simultaneous temperature recordings by GPS collars, thermometers, and a black globe device. (DOC) [file pone.0065972.s002.doc]
