# Supplementary material for: Behavioural Responses to Thermal Conditions Affect Seasonal Mass Change in a Heat-Sensitive Northern Ungulate
Source: PLoS One. 2013 Jun 11;8(6):e65972. doi: 10.1371/journal.pone.0065972 (PMC3679019; doi:10.1371/journal.pone.0065972)

**Supporting Information**

Behavioural responses to thermal conditions affect seasonal mass change in a heat-sensitive northern ungulate – van Beest & Milner

**Figure S3**

Average frequency (%) of summer and winter activity counts (top), step length (middle), and turning angle (bottom) for GPS-collared female moose relocated every hour in southern Norway in 2007-2010. These movement characteristics were used to classify each location as either active or inactive. Step length and turning angle distributions were also used to assign random locations to the used GPS locations, to be used in the RSFs.


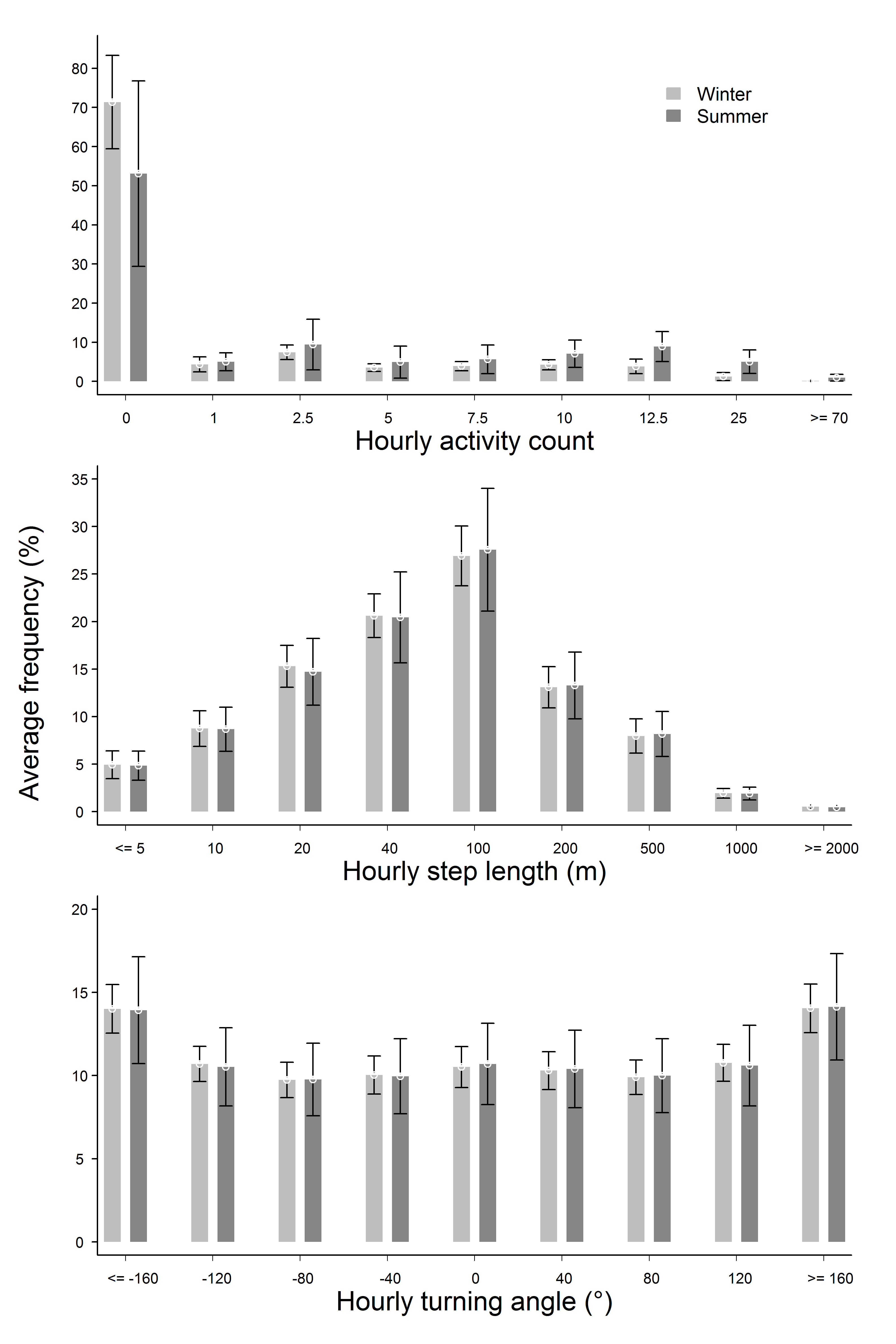

Supplement: Figure S3 — Seasonal movement characteristics of adult female moose in southern Norway. (DOC) [file pone.0065972.s003.doc]
