# Supplementary material for: Behavioural Responses to Thermal Conditions Affect Seasonal Mass Change in a Heat-Sensitive Northern Ungulate
Source: PLoS One. 2013 Jun 11;8(6):e65972. doi: 10.1371/journal.pone.0065972 (PMC3679019; doi:10.1371/journal.pone.0065972)

**Supporting Information**

Behavioural responses to thermal conditions affect seasonal mass change in a heat-sensitive northern ungulate – van Beest & Milner

**Figure S4**

Plot showing the result of the hierarchical cluster and *k*-means analyses for thermoregulatory behaviour by moose during winter. The panel on the left shows a dendrogram with groups of individual moose based on their similarity in thermoregulatory behaviour. Confirming our PCA analyses (see text main article) 4 major thermoregulatory strategies were identified based on *k*-means analyses of the within groups sum of squares (panel on the right).


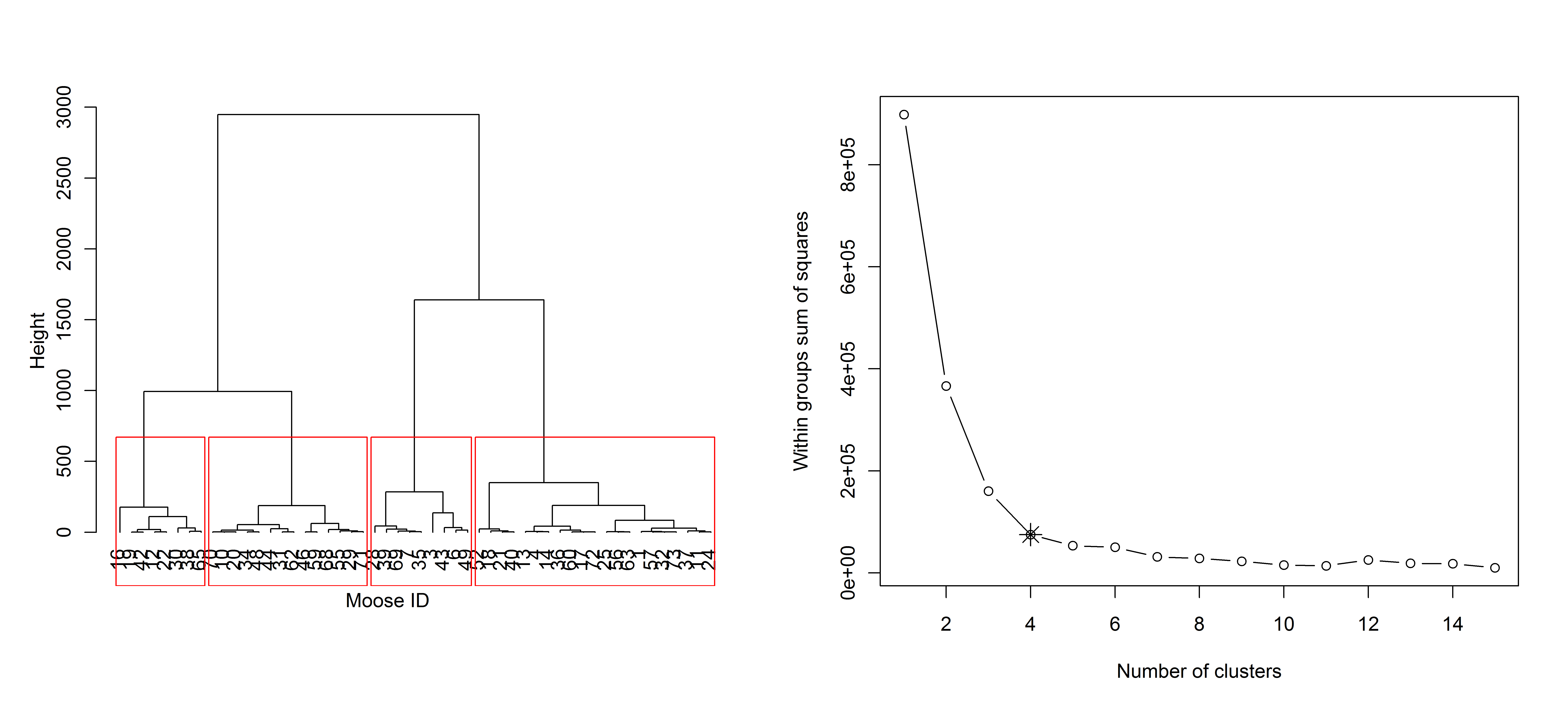

Supplement: Figure S4 — Results of hierarchical cluster and k -means analyses to quantify thermoregulatory strategies by adult female moose during winter. (DOC) [file pone.0065972.s004.doc]
