# Supplementary material for: Behavioural Responses to Thermal Conditions Affect Seasonal Mass Change in a Heat-Sensitive Northern Ungulate
Source: PLoS One. 2013 Jun 11;8(6):e65972. doi: 10.1371/journal.pone.0065972 (PMC3679019; doi:10.1371/journal.pone.0065972)
Supplement: Table S1 — Covariates influencing over-winter mass change for adult female moose in southern Norway. (DOC) [file pone.0065972.s006.doc]

**Supporting Information**

Behavioural responses to thermal conditions affect seasonal mass change in a heat-sensitive northern ungulate – van Beest & Milner

**Table S1**

Overview of the covariates retained in the final model influencing over winter mass change (P ≤ 0.05) for adult female moose (*n* = 52) in southern Norway. High ambient temperature was ≥ 0°C and low ambient temperature was < -5°C. The covariate ‘Number of days marked between weighing events’ was forced into the model to account for temporal variation in reweighing between individuals (see text for details). Also provided are the covariates removed from the full model based on their *F*- and *P*-value.

| **Final covariates** | **β** | **SE** | **df** | ***F*-value** | ***P*-value** | **partial R^2^** |
| --- | --- | --- | --- | --- | --- | --- |
| (Intercept) | 0.022 | 0.101 | 1,44 | 0.220 | 0.827 | - |
| Selection mature conifer forest - high temperature | 0.053 | 0.013 | 1,44 | 15.457 | <0.001 | 0.084 |
| Selection young pine forest - high temperature | -0.038 | 0.009 | 1,44 | 17.532 | <0.001 | 0.095 |
| Selection young spruce forest - high temperature | -0.138 | 0.043 | 1,44 | 10.385 | 0.002 | 0.056 |
| Selection young spruce forest - low temperature | 0.139 | 0.045 | 1,44 | 9.422 | 0.004 | 0.051 |
| Prop. use of feeding stations - low ambient temperature | 0.225 | 0.028 | 1,44 | 64.632 | <0.001 | 0.349 |
| Number of days marked between weighing events | -0.002 | 0.001 | 1,44 | 1.242 | 0.271 | 0.007 |
| Mean altitude (m) used | -0.0001 | 0.00004 | 1,44 | 4.606 | 0.037 | 0.025 |
| **Stepwise removal of covariates** | **Order** | ***F*-value** | ***P*-value** |  |  |  |
| Prop. activity in open mixed forest - high temperature | 1 | 0.003 | 0.955 |  |  |  |
| Selection open mixed forest - low temperature | 2 | 0.014 | 0.906 |  |  |  |
| Prop. activity in young spruce forest - high temperature | 3 | 0.014 | 0.907 |  |  |  |
| Study area | 4 | 0.036 | 0.851 |  |  |  |
| Selection young pine forest - low temperature | 5 | 0.031 | 0.861 |  |  |  |
| Prop. activity in young pine forest - high temperature | 6 | 0.062 | 0.805 |  |  |  |
| Pregnant | 7 | 0.215 | 0.647 |  |  |  |
| Prop. activity in mature conifer forest - high temperature | 8 | 0.131 | 0.720 |  |  |  |
| Year | 9 | 0.300 | 0.744 |  |  |  |
| Prop. activity in deciduous forest - low temperature | 10 | 0.523 | 0.475 |  |  |  |
| Prop. use of feeding stations - high ambient temperature | 11 | 0.800 | 0.378 |  |  |  |
| Prop. activity in young spruce forest - low temperature | 12 | 0.622 | 0.436 |  |  |  |
| Selection mature conifer forest - low temperature | 13 | 0.714 | 0.404 |  |  |  |
| Prop. activity in mature conifer forest - low temperature | 14 | 0.790 | 0.380 |  |  |  |
| Selection other habitat - high temperature | 15 | 0.893 | 0.350 |  |  |  |
| Prop. activity in open mixed forest - low temperature | 16 | 0.529 | 0.472 |  |  |  |
| Prop. activity in other habitat - high temperature | 17 | 0.754 | 0.391 |  |  |  |
| Selection open mixed forest - high temperature | 18 | 1.186 | 0.283 |  |  |  |
| Prop. activity in deciduous forest - high temperature | 19 | 1.997 | 0.165 |  |  |  |
| Selection other habitat - low temperature | 20 | 1.981 | 0.167 |  |  |  |
| Prop. activity in young pine forest - low temperature | 21 | 0.910 | 0.346 |  |  |  |
| Prop. activity in young spruce forest - low temperature | 22 | 2.958 | 0.093 |  |  |  |
