# Supplementary material for: Behavioural Responses to Thermal Conditions Affect Seasonal Mass Change in a Heat-Sensitive Northern Ungulate
Source: PLoS One. 2013 Jun 11;8(6):e65972. doi: 10.1371/journal.pone.0065972 (PMC3679019; doi:10.1371/journal.pone.0065972)
Supplement: Table S2 — Covariates influencing over-summer mass change for adult female moose in southern Norway. (DOC) [file pone.0065972.s007.doc]

**Supporting Information**

Behavioural responses to thermal conditions affect seasonal mass change in a heat-sensitive northern ungulate – van Beest & Milner

**Table S2**

Overview of the covariates retained in the final model influencing over summer mass change (P ≤ 0.05) for adult female moose (*n* = 47) in southern Norway. High ambient temperature was ≥ 20°C and low ambient temperature was < 14°C. The covariate ‘Number of days marked between weighing events’ was forced into the model to account for temporal variation in reweighing between individuals (see text for details). Also provided are the covariates removed from the full model based on their *F*- and *P*-value.

| **Final covariates** | **β** | **SE** | **df** | ***F*-value** | ***P*-value** | **partial R^2^** |
| --- | --- | --- | --- | --- | --- | --- |
| (Intercept) | 0.186 | 0.040 | 1,38 | 4.336 | <0.001 |  |
| Selection mature conifer forest - high temperature | 0.082 | 0.011 | 1,38 | 47.698 | <0.001 | 0.141 |
| Selection young pine forest - high temperature | -0.077 | 0.009 | 1,38 | 97.748 | <0.001 | 0.204 |
| Selection open mixed forest - low temperature | 0.024 | 0.009 | 1,38 | 6.945 | 0.012 | 0.013 |
| Selection young spruce forest - low temperature | 0.036 | 0.013 | 1,38 | 11.708 | 0.002 | 0.021 |
| Prop. activity in young spruce forest - high temperature | -0.055 | 0.020 | 1,38 | 7.782 | 0.008 | 0.017 |
| Number of calves at heel in autumn ^a^ |  |  | 2,38 | 75.364 | <0.001 | 0.349 |
| 1 | -0.122 | 0.015 |  |  |  |  |
| 2 | -0.196 | 0.028 |  |  |  |  |
| Number of days marked between weighing events | 0.0002 | 0.0002 | 1,38 | 0.365 | 0.549 | 0.001 |
| **Stepwise removal of covariates** | **Order** | ***F*-value** | ***P*-value** | P |  |  |
| Selection other habitat - low temperature | 1 | 0.066 | 0.802 |  |  |  |
| Selection open mixed forest - high temperature | 2 | 0.094 | 0.764 |  |  |  |
| Selection other habitat - high temperature | 3 | 0.047 | 0.831 |  |  |  |
| Prop. activity in deciduous forest - high temperature | 4 | 0.220 | 0.645 |  |  |  |
| Prop. activity in young spruce forest - low temperature | 5 | 0.189 | 0.669 |  |  |  |
| Selection young spruce forest - high temperature | 6 | 0.318 | 0.579 |  |  |  |
| Prop. use of feeding stations - low ambient temperature | 7 | 0.158 | 0.695 |  |  |  |
| Prop. activity in deciduous forest - low temperature | 8 | 0.449 | 0.510 |  |  |  |
| Prop. activity in young pine forest - low temperature | 9 | 0.384 | 0.542 |  |  |  |
| Autumn status | 10 | 0.435 | 0.516 |  |  |  |
| Selection young pine forest - low temperature | 11 | 0.609 | 0.443 |  |  |  |
| Prop. activity in mature conifer forest - high temperature | 12 | 0.588 | 0.451 |  |  |  |
| Prop. activity in young pine forest - high temperature | 13 | 0.574 | 0.456 |  |  |  |
| Prop. activity in mature conifer forest - low temperature | 14 | 1.003 | 0.326 |  |  |  |
| Prop. use of feeding stations - high ambient temperature | 15 | 1.635 | 0.212 |  |  |  |
| Selection mature conifer forest - low temperature | 16 | 1.922 | 0.176 |  |  |  |
| Prop. activity in open mixed forest - low temperature | 17 | 1.248 | 0.273 |  |  |  |
| Study area | 18 | 1.186 | 0.283 |  |  |  |
| Prop. activity in open mixed forest - high temperature | 19 | 0.764 | 0.389 |  |  |  |
| Year | 20 | 1.252 | 0.307 |  |  |  |
| Mean altitude (m) used | 21 | 1.350 | 0.253 |  |  |  |
| Prop. activity in other habitat - high temperature | 22 | 3.403 | 0.073 |  |  |  |
| Prop. activity in young spruce forest - high temperature | 23 | 2.746 | 0.106 |  |  |  |
| ^a^ Estimates are in comparison to 0 calves at heel in autumn | | |  |  |  |  |
