# Supplementary material for: Behavioural Responses to Thermal Conditions Affect Seasonal Mass Change in a Heat-Sensitive Northern Ungulate
Source: PLoS One. 2013 Jun 11;8(6):e65972. doi: 10.1371/journal.pone.0065972 (PMC3679019; doi:10.1371/journal.pone.0065972)
Supplement: Text S1 — Evaluating the PCA method with cluster analyses to quantify thermoregulatory strategies. (DOC) [file pone.0065972.s008.doc]

**Supporting Information**

Behavioural responses to thermal conditions affect seasonal mass change in a heat-sensitive northern ungulate – van Beest & Milner

**Text S1. Evaluating PCA with cluster analyses**

To evaluate whether our multivariate gradient analyses accurately classified individuals into thermoregulatory strategies we compared the PCA results with the output of an independent grouping procedure which was based on hierarchical clustering and *k*-means analyses. Determining the variables important to identify clusters is an important area of current research [1]. Inclusion of non-informative covariates can confound underlying cluster structure and it is recommended to consider only covariates known to influence the processes under investigation [2], in our case, thermoregulatory behaviour and seasonal mass change. As such, we first calculated a Euclidean distance dissimilarity matrix based on the values of all covariates retained in the final seasonal mass models. Based on similarity in the Euclidian distance matrix, as determined by Ward’s minimum variance method in the R package hclust, individuals with similar thermoregulatory behaviours were grouped. We produced a dendrogram for each season (Figs S4 and S5) to display the results of the hierarchical clustering procedure, where distance between thermoregulatory behaviour groupings is represented by the height of the lines connecting groups. The optimal number of clusters (i.e. groups of individuals with similar thermoregulatory behaviour) was then determined with *k*-means analysis [1,3], by identifying the point (i.e. clusters) where the change in within groups sum of squares was lowest (Figs S4 and S5).

**References**

1. Van Moorter B, Visscher DR, Jerde CL, Frair JL, Merrill EH (2010) Identifying movement states from location data using cluster analysis. J Wildl Manag 74: 588–594.

2. Kaufman L, Rousseeuw PJ (2005) Finding Groups in Data: An Introduction to Cluster Analysis. Wiley.

3. Contasti AL, Tissier EJ, Johnstone JF, McLoughlin PD (2012) Explaining spatial heterogeneity in population dynamics and genetics from spatial variation in resources for a large herbivore. Plos One 7: e47858. doi:doi:10.1371/journal.pone.0047858.
